# The Evolution of T Cell Depleted Haploidentical Transplantation

**DOI:** 10.3389/fimmu.2019.02769

**Published:** 2019-11-27

**Authors:** Franco Aversa, Antonio Pierini, Loredana Ruggeri, Massimo Fabrizio Martelli, Andrea Velardi

**Affiliations:** ^1^Hematology and Bone Marrow Transplantation Unit, Department of Medicine and Surgery, University of Parma, Parma, Italy; ^2^Division of Hematology and Clinical Immunology, Department of Medicine, University of Perugia, Perugia, Italy

**Keywords:** haploidentical transplantation, T cell depletion, graft vs. host disease, immune reconstitution, graft vs. leukemia effect

## Abstract

Work on bone marrow transplantation from haploidentical donor has been proceeding for over 20 years all over the world and new transplant procedures have been developed. To control both graft rejection and graft vs. host disease, some centers have preferred to enhance the intensity of the conditioning regimens and the post-transplant immune suppression in the absence of graft manipulation; others have concentrated on manipulating the graft in the absence of any additional post-transplant immune suppressive agent. Due to the current high engraftment rates, the low incidence of graft-vs.-host disease and regimen related mortality, transplantation from haploidentical donors have been progressively offered even to elderly patients. Overall, survivals compare favorably with reports on transplants from unrelated donors. Further improvements will come with successful implementation of strategies to enhance post-transplant immune reconstitution and to prevent leukemia relapse.

## Introduction

The great interest in hematopoietic stem cell transplantation (HSCT) from partially matched family donors (haplo-HSCT) arises from several advantages: (1) the donor is immediately available for almost all patients, (2) he/she can be chosen from family members, (3) he/she is highly motivated, and (4) post-transplant donor-derived cellular therapies (such as donor lymphocyte infusions) are easily accessible if needed (1). A recent survey confirmed the numbers of haplo-HSCT performed in Europe continue to increase (2), certainly because of the impressive progress in the clinical, biological, and technical aspects of haplo-HSCT that have been achieved over the past decade. Nowadays, haplo-HSCT is a clinical reality that provides similar outcomes to transplantation from either matched unrelated donors or unrelated cord blood unit (3–5).

Approaches to T-cell depletion have varied greatly in the levels of residual T lymphocytes in the inoculum, intensity of the conditioning regimens and post-transplant immunosuppression (6). On the other hand, interest in unmanipulated or T cell replete haplo-HSCT was reawakened by new strategies for graft-vs.-host disease (GvHD) prophylaxis, such as G-CSF–primed grafts (7, 8), post-transplant rapamycin (9), or high-dose cyclophosphamide (CY) in combination with other immunosuppressive agents (10–13). This latter is mainly based on standard T cell replete stem cell transplants with the aim of making haplo-HSCT as easier as possible. On the contrary, transplant platforms based on T cell depletion rely mainly on graft processing to achieve an ideal graft composition that allows for prevention of rejection, recurrence of leukemia, infections, and GvHD without the need for any further post-transplant immune suppressive treatment. T cell depletion-based grafts require dedicated laboratories and are more expensive than conventional unmanipulated HSCT, especially if combined with adoptive transfer of T cell populations that have been chosen to improve post-transplant immune reconstitution. However, unlike in unmanipulated haplo-HSCT, pharmacologic GvHD prophylaxis and further treatments are not necessary in T cell depleted haplo-HSCT, thus, reducing the need and the cost of supportive care and post-transplant hospitalizations. In recipients of unmanipulated T replete haplo-HSCT followed by post-transplant cyclophosphamide (PTCY), the use of non-ablative conditioning regimen has certainly contributed to reduce non-relapse mortality (NRM). However, non-fatal BK virus-associated hemorrhagic cystitis (HC) occurred in 75% of patients after a busulfan-based conditioning and in 30% of patients after a TBI-based conditioning [reviewed in (14)]. Furthermore, relapse remains a major concern, especially after non-myeloablative conditioning regimens, occurring in ~45–51% of patients (11, 14, 15).

This review will concentrate on the evolution of the T cell depleted (TCD) haplo-HSCT since the main obstacles to its success (lethal GvHD and graft rejection) were overcome in the early 1990s.

## Preventing GvHD and Overcoming Rejection

In the early 1980s the use of soybean lectin agglutination (SBA) followed by rosetting with sheep red blood cells (E-rosette) allowed hematopoietic stem cell engraftment and immune reconstitution in the absence of GvHD. The sedimentation of T cells that spontaneously surrounded sheep red cells (rosettes) made possible the depletion of most of the T lymphocytes that escaped lectin agglutination. Such approach resulted in about a thousand-fold depletion of T-cells (16, 17). Thanks to this technique, patients with severe combined immunodeficiency (SCID) were successfully transplanted with TCD bone marrow graft from a haploidentical donor. T cell depletion facilitated engraftment and ensured no GvHD in these patients (16, 17).

Since the first successful haplo-HSCT in SCID patients, a lectin-based T cell depletion approach has been implemented over the years with great success in hundreds of SCID patients with a very long follow-up. It showed a cure rate that was especially impressive in patients receiving the transplant within the first year after birth (16–19). Following these remarkable results, TCD haplo-HSCT was attempted in patients affected by acute leukemias. In the first patient series such approach failed because of an unacceptable high rate of graft rejection (20, 21). In leukemia patients, anti-donor, recipient type, cytotoxic T-lymphocyte precursors (CTL-p) may survive the conditioning regimen and promote rejection of the donor graft (21–23). Donor T cells that derives from the inoculum eliminate residual host CTL-ps and allow for engraftment in unmanipulated transplants. Such mechanism is not present in TCD transplants. Thus, conditioning regimens that might be conventionally considered sufficient for donor engraftment in unmanipulated transplants are no longer adequate in TCD haplo-HSCT.

The use of a graft containing a “megadose” of TCD hematopoietic progenitor cells was a clinical breakthrough as it overcame such immunological barrier in the absence of an excessive conditioning regimen related toxicity (24, 25). Preclinical studies demonstrated that cells within the human CD34+ hematopoietic stem cell population can specifically neutralize CTL-ps directed against their antigens but not against a third party in mixed lymphocyte reactions. This peculiar ability was called “veto” activity (26–28). The ability of a “megadose” of CD34+ cells to exert *in vivo* “veto” activity and, thus, to facilitate engraftment, was confirmed in a “first in human” clinical trial in Perugia from 1993 to 1995. In this study, TCD haplo-HSCT was performed in 36 acute leukemia patients that received a conditioning regimen with single dose total body irradiation (TBI), cyclophosphamide, anti-thymocyte globulin (ATG), and thiotepa followed by the infusion of ≈10 × 10^6^ CD34+ cells/kg and only 2 × 10^5^ CD3+ cells/kg. This clinical protocol showed robust sustained engraftment in 80% of patients with only 20% of them experiencing GvHD despite the absence of any pharmacologic immune suppressive GvHD prophylaxis (29).

## From Lectins to CD34+ Cell Selection

Following this initial success, efforts have been made to optimize graft processing and reducing the conditioning-related toxicity with the aim to further improve TCD haplo-HSCT outcome.

Grafts containing a median of 2 × 10^5^ CD3+ cells/kg after the lectin-based procedure were associated to a 20% incidence of GvHD. Moreover, in SCID haplo-HSCT, 3 × 10^4^/kg of donor T cells was identified as the threshold for GvHD (17). To further reduce the number of T lymphocytes in the final graft to such level, peripheral blood progenitor cells (PBPCs) mobilized with G-CSF were depleted of T-cells by one round of E-rosetting followed by positive immuno-selection of the CD34+ cells with the Ceprate system (30). This strategy was subsequently abandoned in 1999 when the CliniMACS device (©Miltenyi) allowed for an effective CD34+ cell selection in just one step procedure. This approach is still widely used to date as no other manipulation of leukapheresis products is needed (31).

In 1995 the Perugia group started to use fludarabine instead of cyclophosphamide for the first time in allogeneic HSCT. This modification of the conditioning regimen was based on data from a murine model where conditioning regimens with TBI/cyclophosphamide and TBI/fludarabine provided similar immunosuppression (32). In fact, fludarabine was introduced in order to minimize extra-hematological toxicity and, at the same time, to enhance host immunosuppression (30, 31).

The combination of a fludarabine-based conditioning regimen and the positive selection of the CD34+ cells prevented both rejection and GvHD. However, it is worth noting that *in vivo* persistence of ATG, which was part of the conditioning, may have contributed to the almost complete control of GvHD. At the same time, the conditioning-related toxicity was very low with only a minority of patients developing severe mucositis and no case of veno-occlusive disease of the liver was observed (31).

An analysis of the relapse rate also led to some interesting observations. In fact, despite the absence of GvHD, the leukemia relapse was not increased in these high-risk leukemia patients (31). Several factors may have contributed to eradicate the residual leukemic cells despite the lack of a potent T-cell mediated Graft-vs.-Leukemia (GvL) effect: (1) the intense myeloablation of the conditioning regimen could have reached a deeper reduction of leukemic stem cells in the bone marrow of the patients; (2) the few T cells in the graft may have exerted a subclinical GvL/GvHD effect because they were unopposed by any post-transplant immune suppressive treatment; (3) a strong and T cell independent GvL effect exerted by donor NK cells (33–35). NK-cell function is regulated by a balance of signals mediated by activating and inhibitory receptors (36). NK receptors specific for major histocompatibility complex (MHC) class I molecules, including killer immunoglobulin (Ig)-like receptors (KIR) and the C-type lectin-like CD94/NKG2A, have a role in eradicating residual leukemic cells. NK cells react to the lack of self-HLA expression on allogeneic targets (so-called “missing self-recognition”) (37). In an analysis of 112 patients with high-risk AML, transplantation from NK-alloreactive donors (*n* = 51) was associated with a significantly lower relapse rate in the 61 patients in complete remission (CR) at transplant (3 vs. 47%) (*P* > 0.003) and better event-free survival (EFS) (67 vs. 18%, *P* = 0.02) (38). Results from clinical trials have shown that NK cell alloreactivity is also an effective form of immunotherapy in pediatric acute leukemia (39, 40). The combination of KIR genes define group A haplotype, which has few genes, most of which encoding for inhibitory KIRs, while group B, in addition to inhibitory KIRs, has several genes encoding for activating KIRs (40). In children with acute lymphoid leukemia in complete remission, Oevermann et al. reported a significantly reduced incidence of relapse among the group B haplotype as compared to those of the group A haplotype (33 vs. 64%) (41). Another mechanism that allows for better control of leukemia relapse relies on the use of mothers as donors. In fact, mothers can develop memory T cells against paternal HLA haplotype because of exposure to fetal antigens during pregnancy. This T cell immunity could be responsible for early recognition of such antigens in leukemic cells after transplant resulting in stronger GVL effect when mothers are chosen as donors (42). In addition to the anti-leukemia effect, NK-alloreactive donors carrying KIR2DS1 and/or KIR3DS1 genes also impact on NRM by controlling infections, and so contributing to improve the event-free survival (43). Therefore, the donor-vs.-recipient NK alloreactivity, as predicted by the HLA disparity, should be considered when selecting the optimal donor within the family members.

Apart from the NK alloreactivity, 43% of AML and 30% of ALL patients who were in any CR at transplant survive event-free and GvHD-free with a maximum follow-up of 20 years (31). More recently, the European Group for Blood and Marrow Transplantation (EBMT) performed a retrospective study collecting data from different European centers to analyze the outcome of “mega-dose” haplo-HSCT. This study confirmed the success of the approach by reporting 48% EFS in 266 patients with AML in first CR at the time of transplantation (44).

## Positive Selection of the PBPCs and Post-Tranplant Immunological Reconstitution

While the low number of infused donor T lymphocytes allows for almost full prevention of GvHD in TCD haplo-HSCT, it is also responsible for the major drawback of this approach. In fact, post-transplant T cell immune reconstitution in TCD haplo-HSCT is delayed because it relies only on the expansion of the few T cells infused within the graft and on the development of donor, thymus derived, naïve T cells that occurs several months after transplant in adult patients. Thymus function decays with age and myeloablative conditioning regimen further disrupts thymus and lymphoid structures. These events alter post-transplant T cell dynamics and impede generation of efficient memory T cell immunity (45). Because of the low number of donor T cell in the graft and the additional *in vivo* T cell depletion exerted by the use of ATG in the conditioning regimen, patients that receive TCD haplo-HSCT exhibit a very narrow T-cell repertoire that is responsible for their prolonged susceptibility to life-threatening opportunistic infections (46). In the study by Aversa and colleagues, 27 of 103 patients died because of deadly infections (31). Thus, infection-related mortality was the main cause of transplant failure in this setting.

In this context, it is of note the retrospective analysis that the Swiss Blood Stem Cell Transplantation group made to evaluate the effect on immune reconstitution and incidence of infections in haplo-HSCT from 1998 to 2010. The authors reported 69 transplants that were performed with *ex-vivo* T cell depletion (through CD34 positive selection or CD3/CD19 depletion) or with *in vivo* T cell depletion using anti-CD52 monoclonal antibody alemtuzumab (47). High incidence of life-threatening bacterial, fungal, and viral infections (mostly Cytomegalovirus, CMV) was reported in all these patients. Eventually, the use of alemtuzumab was associated with a higher incidence of CMV reactivations (54 vs. 28%, *p* = 0.015), demonstrating that even *in vivo* T cell depletion should be considered a relevant risk factor in haplo-HSCT (48).

### Improving Immunological Reconstitution After CD34+ Cell Haplo-HSCT

With the aim of diminishing the challenges of life-threatening infections, GvHD, and relapse after TCD haplo-HSCT, various strategies have been investigated over the past decade to facilitate the safe transfer of mismatched T lymphocytes.

The use of post-transplant adoptive transfer of pathogen-specific T lymphocytes represents a possible strategy. A study demonstrated that the infusion of donor derived *ex vivo* selected T cells that were able to clone specifically against Aspergillus or CMV antigens, could control CMV reactivation and reduced detection of galactomannan (49). Interestingly these cells remained pathogen-specific over time after infusion as patients did not develop such infections and did not experience GvHD. Thanks to these promising results, other authors developed similar approaches for the prevention of Adenovirus and Epstein-Barr virus (EBV) infections (50, 51).

Several groups attempted to ameliorate post-transplant immune reconstitution by infusing adoptive T cell immunotherapy with a broad T cell receptor repertoire that resembles physiological conditions. Different approaches have been used to manipulate the graft so that enough T cells could be infused to the patients without causing GvHD.

The group form San Raffaele Institute in Milan, Italy, engineered polyclonal donor T cells to express suicide genes (e.g., the herpes simplex thymidine kinase, TK, and gene). Once infused, these engineered cells could be lysed in case they triggered GvHD by the simple use of Ganciclovir, a widely available anti-viral kinase drug normally used in the treatment of CMV reactivations or diseases (52). One concern is that the mechanism is dependent on cell cycle, thus killing can be delayed and is limited to proliferating cells. Nevertheless, the patients enrolled in this study experienced a low rate of infection-related mortality suggesting functional protection against pathogens (53, 54). Thanks to this experience, it was further possible to understand that TK cell dependent immune reconstitution relies on the thymic generation of T cells derived from differentiated donor hematopoietic precursors (55).

An alternative and more attractive approach is based on the post-transplant infusion of inducible human caspase-9 transgene (iC9) T lymphocytes (56, 57). This technology is based on a cell membrane-permeable small molecule dimerizing drug, AP1903 (also known as Rimiducid). The administration of AP1903 induces dimerization of caspase 9, which activates the terminal effector caspase, caspase 3, with rapid induction of apoptosis. Unlike the HSV-TK–based suicide gene, the iC9 is human derived and has limited immunogenicity and, more important, ganciclovir and related drugs to treat viral infection are allowed without T-cell damage (56). Activation of iC9 produces up to 99% eradication of iC9-expressing T cells within 2 h of a single dose of AP1903 and controls GvHD within 24–48 h. Although administration of AP1903 in patients with GvHD reduces the level of circulating virus-specific iC9-T cells, these cells subsequently recover and *in vivo* antiviral activity is retained.

Another approach aims to *ex vivo* selectively deplete donor-vs.-recipient alloreactive T lymphocytes. T-cell activation is associated with P-glycoprotein pump inhibition, which leads to intracellular accumulation of the rhodamine-derived photosensitizer TH9402, a substrate of this pump Alloreactive T cells preferentially retain the photosensitizer TH9402 and can then be eliminated following exposure to visible light. On the contrary, resting T lymphocytes still exhibit a broad repertoire against infective agents (58–60). More recently, this photodepletion strategy has been tested in a phase I clinical study with aims to find the maximum tolerated dose and to evaluate the safety of allodepleted T-cell immunotherapy (ATIR101), administered in the absence of any additional GvHD prophylaxis, in recipients of CD34+-selected haploidentical HSCT (61). Adults with hematological malignancies were treated with myeloablative TCD haplo-HSCT followed 1 month later by ATIR101 at escalating doses. No patient developed grade III/IV acute GVHD. At 1 year, all nine patients receiving at least one million ATIR101 CD3+ cells/kg did not experience life-threatening infections. After more than 8 years, none of them died because of non-relapse mortality and two thirds of them survive. These promising results set the base for the development of an ongoing phase 3 randomized trial that compares haplo-HSCT + ATIR101 vs. unmanipulated haplo-HSCT + PTCY.

Recently, the group of Perugia employed adoptive transfer of donor CD4+CD25+FOXP3+ regulatory T cells (Tregs) to protect from GvHD that could be caused by the concomitant infusion of high numbers of donor conventional T cells (Tcons) (62, 63). Freshly isolated donor Tregs at a dose of 2 × 10^6^ /kg were given 4 days prior to the infusion of a “megadose” of CD34-positive cells and controlled numbers (0.5–2 × 10^6^/kg) of broad repertoire Tcons, without any post-transplant immunosuppression. GvHD occurred in a minority of the patients proving the effectiveness of the approach despite no post-transplant pharmacologic immune suppressive drug was given to the patients. Moreover, Treg/Tcon adoptive immunotherapy allowed for a fast post-transplant T and B cell immune reconstitution. Diverse naïve and memory T cell subpopulations with a broad T cell receptor repertoire could be early detected and rapidly increased over time. Pathogen-specific CD4+ and CD8+ T cell clones emerged earlier in comparison to patients that received TCD haplo-HSCT with no Treg/Tcon infusions. Treg/Tcon adoptive immunotherapy reduced CMV reactivation episodes with no CMV-related death. More importantly, Treg infusion did not interfere with Tcon mediated GvL effect as leukemia relapse occurred in very few patients despite high-risk diseases (64).

## From Positive to Negative Selection of PBPCs

More recently, the CD34-positive selection technique has been progressively abandoned in favor of a negative selection of the PBPCs with the aim of improving clinical results. In fact, unlike the CD34-positive selected grafts, other immune components, such as NK cells, dendritic cells, and monocytes, are not lost during the negative selection-based procedure and all together these cells contribute to facilitate engraftment, to improve the post-transplant recovery of the anti-infective and anti-leukemia immunity.

### Negative Selection in Children

In the study by Bader et al. (65), grafts were depleted of T and B cells by using CD3- and CD19-coated microbeads and the automated CliniMACS device (Miltenyi Biotec, Germany). Children with acute leukemia received a conditioning that included fludarabine, thiotepa, melphalan, and OKT-3 or ATG. Primary engraftment was achieved in 88% of patients, acute GvHD grade II and III-IV occurred in 20 and 7%, and chronic GvHD in 21%. NRM was 8% at 1 year and 20% at 5 years (65). Using the same T and B cell depletion but a reduced intensity conditioning in 61 adults (median age 46 years), the incidence of grade II–IV acute and chronic GvHD was 46 and 18%, respectively. Non-relapse mortality on Day 100 was 23 and 42% at 2 years. Relapse rate was 31% and OS at 2 years was 28% (66). A major concern with this approach was the high incidence of GvHD.

To overcome this problem, Chaleff et al. recently described a large-scale clinical method using the Miltenyi Biotec CliniMACS^®^ TCR α/β System for the depletion of α/β T lymphocytes from peripheral blood stem cells while retaining all other cells (67). The CliniMACS^®^ TCR α/β System uses murine monoclonal antibodies specific for the T-cell receptor α/β antigen conjugated to biotin in combination with the CliniMACS^®^ Anti-Biotin reagent. The pioneering experience of the Handgretinger's group showed that TCR αβ/CD19 depletion allows a T-cell reduction of 4.5–5 log, which is comparable to CD34+ positive selection (68, 69). It also ensures patients to receive NK cells, monocytes, dendritic cells, and, most important, the TCRγδ+ T lymphocytes. TCRγδ+ T cells appear to exert anti-leukemic activity since they directly recognized stress-induced self-antigens expressed by malignant cells. Strikingly, they do not recognize specific processed peptide antigens as presented on major histocompatibility complex molecules and so are not expected to induce GvHD (70–72).

The first clinical experiences with children transplanted in Tubingen confirmed excellent full-donor engraftment, a rapid early expansion of donor-derived TCRγδ+ T lymphocytes that contributed to a very fast immunological reconstitution (69). Using the same method for graft processing, Locatelli and colleagues in Rome achieved similar results in terms of engraftment, prevention of both acute and chronic GvHD and a rapid recovery of post-transplant immunity in children independently from the conditioning regimen, whether TBI-based (children with leukemia) or chemotherapy-based (children with non-malignant disorders) (73, 74). In 23 children with non-malignant disorders, no cases of visceral acute or chronic GvHD was observed and survival was 91% at 2 years (75). The same group in Rome, starting from encouraging results on a chimeric gene incorporating the death domain of inducible caspase 9 (iC9) (56, 76), has recently launched a phase I/II study enrolling children with either malignant or non-malignant disorders who will receive TCRαβ/CD19-depleted haplo-HSCT, followed by the infusion of titrated numbers of iC9 T cells on day 14 ± 4. These iC9-modified T cells are expected to further improve T cell immune reconstitution without the risk of severe GvHD. In fact, they can be rapidly eliminated by the administration of AP1903, if acute GvHD occurs (77).

TCRγδ+ T cell recovering during the first year after HSCT in 102 patients with acute leukemia correlated with a reduced incidence of infection in the study by Perko et al. (78). Children with an elevated number of TCRγδ+ T cells post-engraftment experienced only viral infection, while low/normal TCRγδ+ T cell group had viral, bacterial and fungal infections. Enhanced TCRγδ+ T cell recovery resulted also in higher EFS rate at 1 year. One can speculate that the following factors may contribute to explain these excellent results: a very fast reconstitution of intestinal mucosa integrity, prompt anti-infective function of TCRγδ+ T cell, and possibly a better balance within gut microbiota (79).

Outcomes of TCRαβ/CD19-depleted haplo-HSCT were evaluated in a cohort of children with chemorefractory AML. The conditioning regimen was designed to include a cytoreduction phase with fludarabine and cytarabine followed by a myeloablative phase with treosulfan and thiotepa. Tocilizumab was given instead ATG in all patients, abatacept in 10 patients. Post-engraftment CD45RA-depleted donor lymphocytes were given prophylactic with or without a hypomethylating agent. Overall results were promising with 95% of patients achieving a complete remission, 18% having a grade II-IV acute GvHD and 23% chronic GvHD. At 2 years, NRM was 9%, relapse rate 42%, event-free and overall survival were 49 and 53%, respectively (80).

More recently, the advantages of this strategy were confirmed in 20 advanced-stage Sickle Cell Disease (SCD) patients (children and adults, median age 15 years). Conditioning consisted of ATG, thiotepa, fludarabine, and treosulfan. Two patients succumbed to a CMV pneumonitis and a macrophage activation syndrome. One patient requires renal replacement therapy because of BK virus nephritis. None developed grade III-IV acute GvHD. At a median follow-up of 21 (range 9–62) months, 90% of these high-risk patients survive showing the feasibility, safety, and efficacy of TCD haplo-HSCT also for advanced stage SCD patients (81).

### Negative Selection in Adults

This approach was recently tested in 59 adult patients (median age 48 years, range 19–74) with hematological malignancies, mostly acute leukemias (82). At the time of transplant, 35 (60%) were in first or later remission and 24 (40%) in advanced phase. All patients were conditioned with a chemotherapy-based regimen that included ATG, treosulfan, fludarabine, and thiotepa. No additional pharmacologic prophylaxis for GvHD was given after transplantation and to minimize the *in-vivo* T cell depletion, ATG was given at a median of 10 days before the graft infusion. A full donor sustained engraftment was achieved in 56/59 (95%) patients. Severe GvHD occurred in two patients who subsequently died from complications due to the GvHD itself and its treatment. One of them had received the highest dose of αβ+ T cells (3.7 × 10^5^/kg). Skin limited grade II acute GvHD was observed in 8 patients who responded rapidly to steroids. Only two patients have so far developed chronic GvHD that recovered completely after steroid and cyclosporine treatment. Interestingly, also in these adults with high-risk hematological malignancies, numbers and functions of the immune system recovered very soon after the engraftment. Naïve and memory T-cell subsets increased significantly over the first year after transplantation. B-cell reconstitution was rapid and immunoglobulin serum levels normalized within 3 months. The quality of the immunological reconstitution allowed a good control of the CMV reactivation with no cases occurring after the first 2 months after transplantation. In two patients, CMV reactivation was associated with a significant expansion of pathogen-specific CD8+ T cells that contributed to clear viral load spontaneously. Relapse of the underlying disease was the main cause of death in 16/59 patients; 15 patients died without relapsing, 11 of them from infections. Age at time of transplantation was a significant risk factor for NRM. Three of the 28 patients (10.7%) aged ≤ 48 years and 12 of the 31 (38.7%) over 48 years of age have so far died from non-relapsing causes. Cumulative incidence of NRM at 2 years was 18% for patients aged ≤ 48 years and 47% for those over 48 years of age (*p* = 0.011). At a median of 27 months (range 1–62), 30 patients (50.8%) survive.

Similar results have been recently reported by a team in Turkey in 34 adult patients with either AML (*n* = 24) or ALL (*n* = 10) (83). Conditioning regimen consisted of thiotepa, melphalan, fludarabine, and ATG. Full donor chimerism was achieved in 31/34 patients. Overall, four patients developed severe GvHD (2 acute, 2 chronic). A low NRM (11.7%) at day 100 was attributed to a rapid T-cell reconstitution. Relapse still remained the main cause of death (56.3%). At 1 year, 42% of the patients survive disease-free.

## Conclusions

Haplo-HSCT is an attractive treatment for patients with high-risk hematological malignancies lacking a well-matched unrelated donor and who require a HSCT urgently. Today, rejection and GvHD are no longer major issues and a recent registry-based study of the EBMT confirmed that outcomes of TCD haplo-HSCT have improved over time reflecting gaining experience, better selection of the donor-recipient pairs, evolution of the conditioning regimens, better supportive care, and treatment options for infections complications, that remain the main cause of death in this setting (84).

Years of research have taken us from a haplo-HSCT containing a megadose of CD34-positive cells and very few donor T lymphocytes to a new “designed” graft containing a megadose of selectively depleted PBPCs and also different types of non-alloreactive immune cells meant to improve immune recovery in the absence of any additional post-transplant immune suppressive prophylaxis of the GvHD (Table 1).

**Table 1:** Evolution of T cell depleted allogeneic HSCT.

**Years**	**Technique**	**Donor**	**Disease**
1980s	TCD BM cells by SBA+E-rosetting	Matched	HMs
		Haplo	SCID
1990s	TCD PBPCs by positive selection of the CD34+ cells using the CliniMACS device	Matched	HMs, N-MHs
		Haplo	HMs, N-MHs
Early 2000s	CD3/CD19 selection of the PBPCs	Haplo	HMs, N-MHs
Late 2000s	CD34+ cell selection + Tregs/Tcons	Haplo	HMs
Late 2000s	Selective depletion of TCRαβ/CD19 cells in some center followed by cell therapy with HSTK-engineered lymphocytes, photodynamic purged T cells, iC9	Matched Haplo	HMs, N-MHs HMs, N-MHs

*TCD, T Cell Depleted; BM, Bone Marrow; SBA, SoyBean Agglutinin; PBPCs, Peripheral Blood Progenitor Cells; Tregs, T regulatory cells; Tcons, Conventional T cells; HMs, Hematological Malignancies; N-MHs, Non-Malignant Hematologic diseases; HSTK, Herpes Simplex Thymidine Kinase; iC9, inducible Caspase-9*.

In this way, an innovative strategy has been recently designed by the Perugia group using a Total Marrow/Total Lymphoid Irradiation-based conditioning regimen followed by the infusion of TCD Treg/Tcon haplo-HSCT to treat elderly patients (aged 55–68 years) with acute myeloid leukemia. None of the first 14 transplanted patients have so far relapsed (85). On the other hand, in a recent retrospective cohort study by Solomon et al., recurrent disease was the main cause of death, in particular in patients aged 55–70 years for whom a RIC protocol was adopted to minimize the transplant-related toxicity (86).

In conclusion, we believe that TCD is still valid in haplo-HSCT for the following main reasons: (a) it guarantees patients to have a good quality of life in the absence of GvHD, in particular in the elderly who, due to the age-related comorbidities, are less able to tolerate GvHD and its treatments; (b) it provides a safer platform for advanced treatment with infusions of TCR-transgenic T-cells, genetically modified redirected NK cells or donor T cells bearing chimeric antigen receptor (CAR-T) to reduce, or even abrogate, the risk of recurrence of the underlying disease.

## Author Contributions

FA and AP wrote the manuscript. LR, MM, and AV reviewed and edited the manuscript.

### Conflict of Interest

The authors declare that the research was conducted in the absence of any commercial or financial relationships that could be construed as a potential conflict of interest.
